# Virtual Care Provision and Emergency Department Use Among Children and Youth

**DOI:** 10.1001/jamanetworkopen.2025.50532

**Published:** 2025-12-18

**Authors:** Gabrielle Freire, Eyal Cohen, Therese A. Stukel, Isobel Sharpe, Xuesong Wang, Daniel Rosenfield, Azmina Altaf, Astrid Guttmann, Monica Kopec, Natasha R. Saunders

**Affiliations:** 1 The Hospital for Sick Children, Toronto, Canada; 2 Department of Pediatrics, Temerty Faculty of Medicine, University of Toronto, Toronto, Canada; 3 Child Health Evaluative Sciences, SickKids Research Institute, Toronto, Canada; 4 ICES, Toronto, Canada; 5 Institute of Health Policy, Management and Evaluation, The University of Toronto, Toronto, Canada; 6 Edwin S.H. Leong Centre for Healthy Children, University of Toronto, Toronto, Canada

## Abstract

**Importance:**

Use of virtual care has rapidly expanded, but its impacts in pediatric emergency department use remain unclear.

**Objective:**

To assess whether modality of primary care visits (in-person visit or virtual visit) is associated with subsequent emergency department (ED) use in acutely ill children.

**Design, Setting, and Participants:**

Population-based cohort study using routinely collected administrative data of children and youth (age 0-17 years) in Ontario, Canada. Participants were children who had a primary care sick visit between September 2020 and March 2024. One visit per child was randomly selected during the study period and children were categorized by age group (aged <3 months, 3 months to <2 years, and 2-17 years).

**Exposures:**

Primary care sick visit modality identified from physician billing codes.

**Main Outcomes and Measures:**

The primary outcome was an ED visit within 3 days of the primary care visit. Secondary outcomes included (1) ED visits leading to hospitalization or death, (2) high-acuity ED visits, and (3) low-acuity ED visits. Age-stratified multivariable logistic generalized estimating equation models were used to estimate the association between modality of the primary care visit and each outcome, clustering patients within physicians and adjusting for sociodemographic, clinical, and health system factors.

**Results:**

Participants included 2 608 503 unique children with a sick visit (132 352 [5.1%] aged <3 months; 282 720 [10.8%] aged 3 months to <2 years; 2 608 503 [84.1%] aged 2-17 years). Virtual visits comprised 719 119 (27.6%) of sick visits. Children aged 3 months to less than 2 years had an increased adjusted risk of a subsequent ED visit when seen virtually compared with in person (4.4% vs 3.5%; adjusted risk ratio [ARR], 1.49; 95% CI, 1.41-1.57) as did children aged 2 to 17 years (2.2% vs 2.4%; ARR, 1.19; 95% CI, 1.15-1.24). Children aged 3 months to less than 2 years and aged 2 to 17 years seen virtually had a lower risk of ED visits leading to hospitalization or death, similar risk of high-acuity ED visit, and higher risk of low-acuity ED visits. Children aged less than 3 months seen virtually had a similar risk of subsequent ED visits to those seen in person.

**Conclusions and Relevance:**

In this population-based cohort study of Ontario children and youth, while emergency department visits following a primary sick visit were infrequent, virtual sick visits to primary care were associated with a small increase in emergency department use in children aged 3 months or older, especially low-acuity emergency department visits. Judicious use of virtual primary care to manage acute illness in children is warranted.

## Key Points

**Question:**

Is the modality of primary care sick visits (virtual visit vs in-person visit) associated with subsequent emergency department (ED) use in children?

**Findings:**

In this population-based cohort study of data from 2 608 503 unique children, virtual sick visits were associated with a modestly higher risk of ED use within 3 days, particularly for low-acuity visits, among children aged 3 months to 17 years. No difference in ED use was observed among infants aged less than 3 months.

**Meaning:**

The findings of this study suggest that a cautious approach to the use of virtual care for managing acute pediatric illness may be warranted.

## Teaser

This cohort study assesses whether the modality of primary care visits (in-person visit or virtual visit) is associated with subsequent emergency department use in acutely ill children.

## Introduction

In recent years, there has been an increase in the use of telemedicine and a decrease in in-person consultations for primary care and emergency departments (ED).^1,2,3,4^ Use of virtual care increased substantially out of necessity in 2020, and virtual care has become a permanent and routine modality offered in many jurisdictions.^5,6^ In Ontario, Canada, 13% of primary care continues to be delivered virtually.^2,5,7^

Virtual services provide the advantage of patient-reported convenience and accessibility^8,9,10,11,12,13,14^ but also have drawbacks, such as the lack of physical examination in virtual encounters, which can impact accurate diagnosis and management of certain conditions.^12,15^ The broader repercussions of virtual care services in health care systems remain unclear.^16^ Some studies, limited to adult populations, have reported increased rates of ED, acute care, and repeat visits among patients seen virtually compared with in-person visits.^8,17,18,19^ Conversely, other studies have reported no association between virtual visits and subsequent ED use.^1^ Studies of virtual care can inform the understanding of the scope of encounters and contexts for which virtual care is appropriate.

While there is a growing body of literature assessing the merits of virtual care in adult care, its benefits and harms among children and adolescents are not well studied. Compared with adults, children may have more subtle clinical signs not easily identifiable on video or phone (eg, middle ear effusion, poor skin turgor, or decreased responsiveness to handling), and an in-person examination may have advantages. Thus, the virtues of virtual care identified in studies with an adult population may not apply to children, and a better understanding of how this modality contributes to health outcomes for children is needed to inform digital health policies.

To fill this knowledge gap, we sought to determine, among children and adolescents (aged 0-17 years) seeking primary care for an acute illness, whether exposure to virtual care is associated with an increased risk of a subsequent ED visit within 3 days of the index primary care sick visit compared with in-person care. We also aimed to assess the association for sick visits with (1) subsequent hospitalization or death, (2) subsequent high-acuity ED visit, and (3) subsequent low-acuity ED visit. We hypothesized that children who had a virtual consultation to a primary care practitioner for an acute illness would be at higher risk of a subsequent ED visit than those seen in person, a lower risk of hospitalization or death and high-acuity ED visits, and a higher risk of low-acuity ED visits.

## Methods

### Study Design

We conducted a population-based cohort study using routinely collected administrative data of children and youth (aged 0-17 years) in Ontario, Canada. We accessed and analyzed data at ICES (formerly the Institute for Clinical Evaluative Sciences), an independent nonprofit research institute whose legal status under Ontario’s health information privacy law allows it to collect and analyze health care and demographic data, without consent, for health system evaluation and improvement. Health and administrative data sources, including all outpatient visits, ED visits, and hospitalizations, as well as sociodemographic data are linked through unique encoded identifiers. The use of these data was authorized under section 45 of Ontario’s Personal Health Information Privacy Act, which does not require review by a Research Ethics Board or individual patient consent. This study followed the Reporting of Studies Conducted Using Observational Routinely-Collected Data (RECORD) extension of the Strengthening the Reporting of Observational Studies in Epidemiology (STROBE) reporting guideline for cohort studies.^20^

### Data Sources

We used the Ontario Health Insurance Plan physician claims database for insured services to identify outpatient visits. We retrieved ED visit data from the National Ambulatory Care Reporting System and hospital discharges from the Canadian Institute for Health Information Discharge Abstract Database. We identified clinician-level characteristics from the Corporate Provider Database. We used the Ontario health care registry, the Registered Persons Database, and 2016 Canadian Census to extract sociodemographic variables. Lastly, we used the Statistics Canada Postal Code Conversation File linked to the Registered Persons Database to determine rurality. A description of the data sources can be found in eTable 1 in Supplement 1.

### Setting and Population

We conducted this study in Ontario, Canada, where health care is delivered through a single-payer model, managed provincially. All children and youth living in Ontario and eligible for provincial health insurance who had a visit to a primary care practitioner (ie, family physician or pediatrician) in a nonhospital setting from September 1, 2020, to March 31, 2024, were eligible for inclusion. We included only sick visit encounters for physical illness, defined as any encounter in which care did not involve a well-child visit^21^ or mental or behavioral health concern. We excluded encounters for mental or behavioral health concerns because physical examinations are not routinely required in this context (eTable 2 in Supplement 1). We excluded individuals who had invalid health card numbers or who had missing date of birth or sex. We also excluded sick visits to physicians with less than 10 sick child visits in the study period for children aged less than 2 years and sick visits to physicians with less than 30 total sick child visits for children aged 2 to 17 years, as these physicians treat very few children within each age strata. Recognizing that children may have had multiple episodes of illness within the study period and multiple sick visits within each episode, we randomly selected a single episode of illness per child for analysis. We stratified the cohort by clinically important age groups (0 to <3 months [young infants], 3 months to <2 years [infants and young children], and 2 to 17 years [children and adolescents]).^22,23^

### Variables

#### Exposures

The main exposure was modality of the sick visit, categorized as either an in-person visit or a virtual visit. Virtual visits included both audio-only and audio-video encounters. We ascertained this using virtual billing fee codes that were introduced during the COVID-19 pandemic (March 2020) to track virtual care provision and ultimately made permanent for primary care clinicians (eTable 2 in Supplement 1). For children and adolescents with multiple sick visits to primary care in the first 2 days of an illness episode, the most recent visit determined the start of the observation period (eFigure in Supplement 1). When children had multiple visits that included both an in-person and virtual modality during the episode of illness, we considered this an in-person visit.

#### Outcomes

The primary outcome was the occurrence of any unscheduled ED visit within 3 calendar days of the index sick visit. We measured the following secondary outcomes: unscheduled ED visits of low acuity (Canadian Triage Acuity Scale [CTAS] score 4-5),^22,23^ unscheduled ED visits of high acuity (CTAS score 1-2), and subsequent hospitalization or in-hospital death within 3 calendar days of the index sick visit (eFigure in Supplement 1).

#### Covariates

We controlled for the following covariates: patient-related sociodemographic factors (sex, age, neighborhood-level material resources quintile,^24^ rural or urban residence [rural population size <10 000 residents]), clinical factors (diagnosis of complex chronic condition [eTable 3 in Supplement 1] over a lifetime look-back period,^25^ and type of usual practitioner of care [UPC]).^25^ We assigned UPC status to the physician who performed the most well-child visits in a 4-year look-back window from the index visit. In the event that physicians performed an equal number, the physician who provided the most recent well-child visit was assigned as UPC. If there was no well-child visit in that period and the child was formally rostered to a family physician, we assigned UPC status to that practitioner.^26^ If there were no well-child visits in that period, and the child was not rostered to a family physician, they were assigned a no UPC status. We categorized UPC as enrolled family physician, unenrolled family physician, pediatrician, other (eg, nurse practitioner), or no UPC.^27^ We collected data relating to the index sick visit, including whether the index practitioner had seen the child for any reason in the 2 years preceding the index visit (indicating an active preexisting patient-clinician relationship). We recorded the total number of sick visits for each child during the study period and the number of outpatient sick visits during each illness episode.

### Statistical Analysis

For each age strata, we reported descriptive statistics with standardized differences (SD) to assess balance in covariates across exposure groups with differences of less than 0.1 indicating balance across groups.^28^ Separately for each age strata, we used multivariable Poisson generalized estimating equation models, which provided adjusted risk ratios (ARRs) and 95% CIs, to estimate the association between modality of the sick-child visit and the risk of an ED visit, adjusting for sex, material resource quintile, rurality, chronic complex conditions, number of visits within the sick episode, index practitioner preexisting relationship, and type of UPC, while clustering patients within physicians.^29^ Results were considered statistically significant if the 95% CI did not include 1.0. We also adjusted for age within the group aged 2 to 17 years, as it spanned a wider spectrum of developmental stages and presentations. We used the same models to evaluate the association between sick visit modality and our secondary outcomes.

Because children with multiple visits within the same illness episode likely differ from children with a single visit, we performed a sensitivity analysis excluding children with multiple visits within the same illness episode. To assess whether our visit modality assignment process for children with multiple visits could have introduced bias, we performed a sensitivity analysis assigning visit modality based on the modality of first visit, regardless of subsequent visit modalities. All statistical analyses were performed using SAS version 9.3 (SAS Institute).

## Results

### Characteristics of Cohort

**Figure 1.**
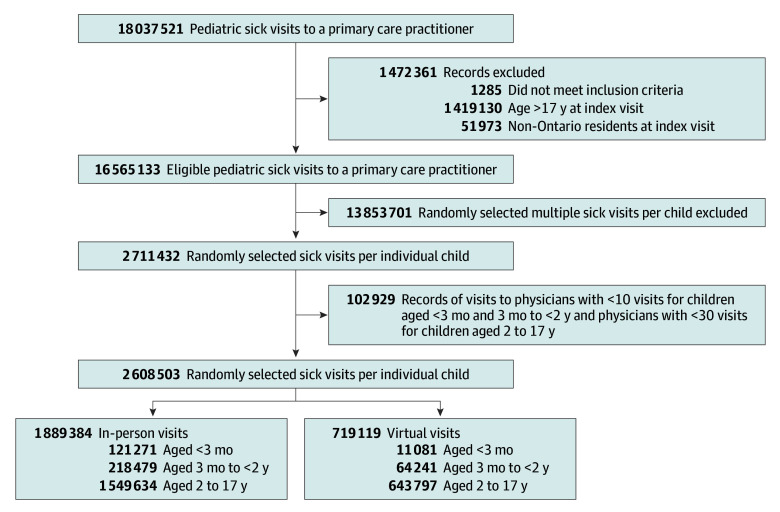
Outpatient Sick Visits to Primary Care for Ontario Children, September 2020 to March 2024

We identified 2 608 503 unique children with sick visits, of which 719 119 (27.6%) were virtual (Figure 1). There were 132 352 (5.1%) children aged less than 3 months, 282 720 (10.8%) children aged 3 months to less than 2 years, and 2 193 431 (84.1%) children aged 2 to 17 years. Across age groups, 1 290 234 children with sick visits were female (49.5%) and 187 957 (7.2%) resided in rural areas. All age groups had a stable distribution across material resource quintiles (from 18.2% in quintile 5 to 20.5% in quintile 1). Among infants aged less than 3 months, 92 976 of 132 352 children (70.2%) had no UPC, whereas the rates were 6.5% (18 470 of 282 720 children) for children aged 3 months to less than 2 years and 10.5% (229 451 of 2 193 431 children) for children aged 2 to 17 years (Table 1).

**Table 1.** Baseline Characteristics of Children With Sick Visits by Modality and Age Group

Characteristic	Sick child visit by age group, No. (%)								
	<3 mo			3 mo to <2 y			2 to 17 y		
	In-person visits (n = 121 271)	Virtual visits (n = 11 081)	SD	In-person visits (n = 218 479)	Virtual visits (n = 64 241)	SD	In-person visits (n = 1 549 634)	Virtual visits (n = 643 797)	SD
Age at index visit, median (IQR), y	0 (0-0)	0 (0-0)	0.00	0 (0-1)	0 (0-1)	0.06	10 (6-14)	11 (6-15)	0.16
Sex									
Female	57 661 (47.5)	5357 (48.3)	0.02	104 893 (48.0)	31 328 (48.8)	0.02	761 883 (49.2)	329 112 (51.1)	0.04
Male	63 610 (52.5)	5724 (51.7)		113 586 (52.0)	32 913 (51.2)		787 751 (50.8)	314 685 (48.9)	
Material resources quintile									
1 (Least marginalized)	22 518 (18.6)	2260 (20.4)	0.05	40 491 (18.5)	12 961 (20.2)	0.04	314 305 (20.3)	137 893 (21.4)	0.03
2	26 349 (21.7)	2646 (23.9)	0.05	49 272 (22.6)	14 851 (23.1)	0.01	362 020 (23.4)	155 750 (24.2)	0.02
3	24 131 (19.9)	2305 (20.8)	0.02	44 817 (20.5)	13 350 (20.8)	0.01	318 648 (20.6)	134 903(21.0)	0.01
4	22 210 (18.3)	2034 (18.4)	0.00	39 423 (18.0)	11 343 (17.7)	0.01	258 211 (16.7)	104 868 (16.3)	0.01
5 (Most marginalized)	24 686 (20.4)	1785 (16.1)	0.11	42 959 (19.7)	11 438 (17.8)	0.05	286 686 (18.5)	107 470 (16.7)	0.05
Missing	1377 (1.1)	51 (0.5)	0.08	1517 (0.7)	298 (0.5)	0.03	9764 (0.6)	2913 (0.5)	0.02
Rural residence	7703 (6.4)	778 (7.0)	0.03	17 152 (7.9)	4403 (6.9)	0.04	115 869 (7.5)	42 052 (6.5)	0.04
Chronic complex conditions	5451 (4.5)	477 (4.3)	0.01	13 752 (6.3)	3535 (5.5)	0.03	96 526 (6.2)	41 957 (6.5)	0.01
Type of UPC^a^									
Family physician, enrolled	27 408 (22.6)	4430 (40.0)	0.38	134 939 (61.8)	41 391 (64.4)	0.06	1 075 613 (69.4)	477 389 (74.2)	0.11
Family physician, unenrolled	2409 (2.0)	273 (2.5)	0.03	9494 (4.3)	2073 (3.2)	0.06	43 478 (2.8)	17 372 (2.7)	0.01
Pediatrician	3694 (3.0)	1129 (10.2)	0.29	57 895 (26.5)	18 026 (28.1)	0.04	246 947 (15.9)	98 940 (15.4)	0.02
Other	31 (<0.1)	≤5^a^ (<0.1)	0.01	375 (0.2)	57 (0.1)	0.02	3198 (0.2)	1043 (0.2)	0.01
No UPC	87 729 (72.3)	5244-5249^a^ (47.4)	0.53	15 776 (7.2)	2694 (4.2)	0.13	180 398 (11.6)	49 053 (7.6)	0.14
Index practitioner was UPC	11 673 (9.6)	2716 (24.5)	0.40	97 773 (44.8)	36 338 (56.6)	0.24	613 160 (39.6)	328 905 (51.1)	0.23
Index practitioner saw child in the last 2 y	47 614 (39.3)	6931 (62.5)	0.48	141 827 (64.9)	48 726 (75.8)	0.24	825 442 (53.3)	441 756 (68.6)	0.32
No. of outpatient visits in sick episode, median (IQR)	1 (1-1)	1 (1-1)	0.38	1 (1-1)	1 (1-1)	0.05	1 (1-1)	1 (1-1)	0.08

Abbreviations: SD, standardized difference; UPC, usual practitioner of care.

^a^ Small cells containing 5 or fewer patients were suppressed to maintain confidentiality in accordance with ICES policy.

### Characteristics of ED Visits

Of 2 608 503 sick visits to primary care, 66 048 (2.5%) resulted in a subsequent ED visit: aged less than 3 months, 3667 of 132 352 visits (2.8%); aged 3 months to less than 2 years, 10 487 of 282 720 visits (3.7%); and aged 2 to 17 years, 51 894 of 2 193 431 visits (2.4%). Of 2 608 503 sick visits to primary care, 5710 (0.2%) resulted in hospitalization or death, 15 316 (0.6%) visits resulted in a high-acuity (CTAS 1-2) ED visits, and 19 462 (0.7%) resulted in a low-acuity (CTAS 4-5) ED visit (Table 2).

**Table 2.** Emergency Department Visits After Primary Care Sick Episodes by Modality and Age Group

Visit characteristic	Visit modality by age group, No. (%)					
	<3 mo		3 mo to <2 y		2 to 17 y	
	In-person visits (n = 121 271)	Virtual visits (n = 11 081)	In-person visits (n = 218 479)	Virtual visits (n = 64 241)	In-person visits (n = 1 549 634)	Virtual visits (n = 643 797)
Any ED visit	3345 (2.8)	322 (2.9)	7636 (3.5)	2851 (4.4)	37 957 (2.4)	13 937 (2.2)
Hospital admission or death	747 (0.6)	59 (0.5)	942 (0.4)	137 (0.2)	3186 (0.2)	639 (0.1)
ED visit acuity						
High (CTAS 1-2)	1813 (1.5)	153 (1.4)	2298 (1.1)	624 (1.0)	7804 (0.5)	2624 (0.4)
Urgent (CTAS 3)	1169 (1.0)	140 (1.3)	3539 (1.6)	1620 (2.5)	17 350 (1.1)	7313 (1.1)
Low (CTAS 4-5)	354 (0.3)	26-30^a^	1779 (0.8)	601-605^a^	12 710 (0.8)	3986 (0.6)
Unknown	9 (<0.1)	≤5^a^	20 (<0.1)	≤5^a^	93 (<0.1)	14 (0)

Abbreviations: CTAS, Canadian Triage and Acuity Scale; ED, emergency department.

^a^ Small cells containing 5 or fewer patients were suppressed to maintain confidentiality in accordance with ICES policy.

### Primary Analysis

**Figure 2.**
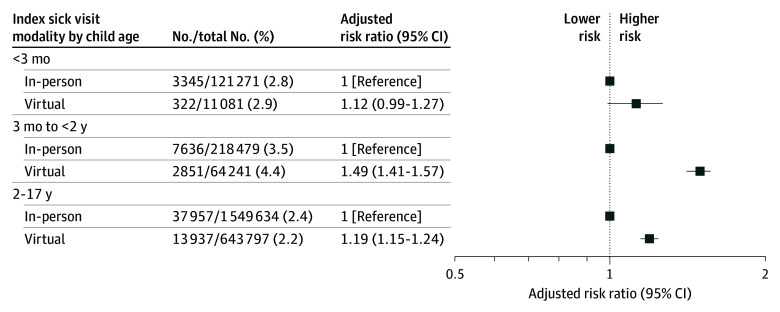
Adjusted Risk Ratios for Emergency Department Visits After Primary Care Sick Visits for Children Models adjusted for patient sex, chronic complex conditions, material resources quintile, rurality, usual practitioner of care (specialty), index practitioner preexisting relationship, number of outpatient visits within the sick episode, and age at the index visit (for the aged 2-17 years group only).

After adjustment, there was no significant difference in subsequent ED visit when children aged less than 3 months were seen virtually compared with in person (2.9% [virtual] vs 2.8% [in person, referent], adjusted risk ratio [ARR], 1.12; 95% CI, 0.99-1.27) (Figure 2). Children aged 3 months to less than 2 years had an increased risk of subsequent ED visit when seen virtually compared with in person (4.4% vs 3.5%; ARR, 1.49; 95% CI 1.41-1.57). Children aged 2 to 17 years also had an increased risk of subsequent ED visit when seen virtually compared with in person (2.2% vs 2.4%; ARR, 1.19; 95% CI, 1.15-1.24).

### Secondary Analyses

**Figure 3.**
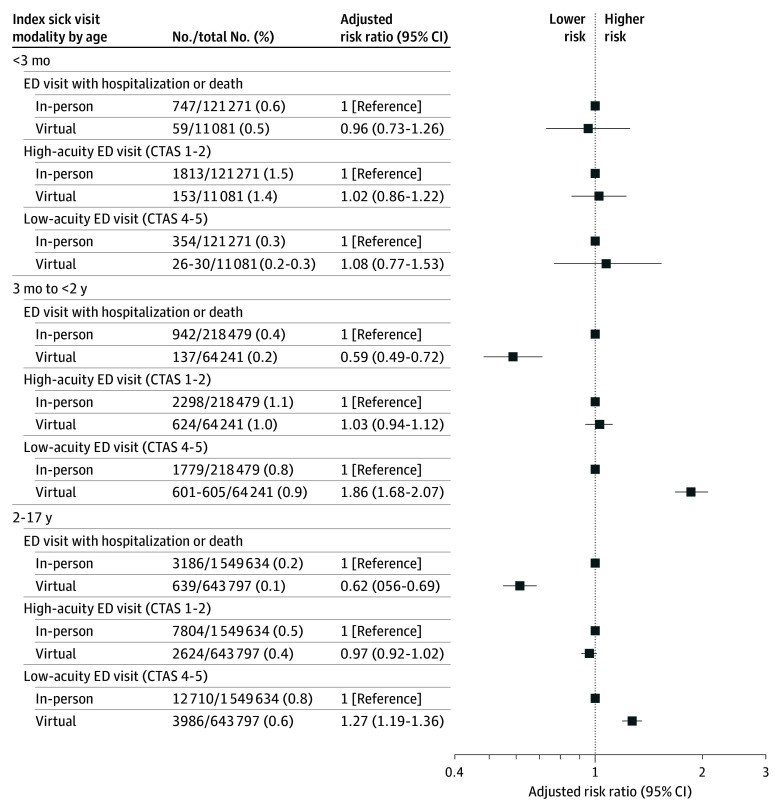
Adjusted Risk Ratios for Severity of Emergency Department Visits After Primary Care Sick Visits Models adjusted for patient sex, chronic complex conditions, material resources quintile, rurality, usual practitioner of care (specialty), index practitioner preexisting relationship, number of outpatient visits within the sick episode, and age at the index visit (for the aged 2-17 years group only). Numbers in the second column given as a range were provided to prevent back-calculation of small cells as per institutional policy.

After adjustment, children aged less than 3 months seen virtually had no difference in subsequent hospitalization or death or high-acuity or low-acuity ED visit compared with those seen in person (Figure 3). In contrast, those aged 3 months or older seen virtually had a lower risk of subsequent hospitalization or death compared with in person (aged 3 months to <2 years: 0.2% vs 0.4%, ARR, 0.59; 95% CI, 0.49-0.72; aged 2 to 17 years: 0.1% vs 0.2%; ARR, 0.62; 95% CI, 0.56-0.69), no difference in high-acuity ED visit, and higher risk of low-acuity ED visits (aged 3 months to <2 years: 0.9% vs 0.8%; ARR, 1.86; 95% CI, 1.68-2.07; aged 2 to 17 years: 0.6% vs 0.8%; ARR, 1.27; 95% CI, 1.19-1.36).

### Sensitivity Analyses

When excluding children with multiple outpatient visits within their sick episode, children aged less than 3 months seen virtually were at increased risk of subsequent ED visit (ARR, 1.16; 95% CI, 1.02-1.32) (eTable 4 in Supplement 1). When defining visit modality based on the first outpatient visit within each sick episode, children aged less than 3 months seen virtually were at increased risk of subsequent ED visits (ARR, 1.24; 95% CI, 1.10-1.39) (eTable 5 in Supplement 1). Results for children aged greater than 3 months to less than 2 years and aged 2 to 17 years were unchanged from the main analysis.

## Discussion

In this large cohort study of children seen in primary care for a sick episode, we observed a small but important increased risk of subsequent ED visit among those seen virtually compared with those seen in person, particularly for children aged 3 months to less than 2 years. Virtual visits were associated with a higher risk of low-acuity ED visits but a lower risk of hospitalization or death. While most (>97%) children seen in primary care for a sick episode do not ultimately visit an ED within 3 days, these findings suggest that despite the safety, convenience, and accessibility offered by virtual care, in-person visits may offer a small advantage in adequately addressing a child’s health concerns, thus alleviating the need to go to an ED for further assessment and reducing the burden on already overcrowded EDs.

To our knowledge, this study is the first to report on the association of virtual care and subsequent ED use for sick visits in children. Existing reports are limited to adult populations and suggest that patients are as likely or less likely to visit ED following virtual encounters.^1,17,30^ A study in an adult population also suggests that virtual encounters are more effective if performed by a patient’s UPC.^30^ In Ontario, this evidence has informed the development of policies that limit remuneration of virtual care within the primary care context to those in which a preexisting relationship exists between patients and clinicians. These polices were applied to all virtual encounters, including those involving children. However, our findings contrast with this study^30^ of an adult population and suggest that policies specific to virtual care delivery in children could be considered, especially for conditions in which physical examination is an important part of diagnosis (eg, otitis media, lower-respiratory tract infection, or dehydration).

A few important differences between adults and children may explain why our findings differ from existing studies of adult population. First, developmental limitations in communication force clinicians to seek proxies from parents when taking a history (eg, irritability and crying as a proxy for pain in younger children) and make the physical examination a more important part of clinical assessments in children. In some cases, clinical signs can be too subtle to appreciate on video (eg, responsiveness to handling, work of breathing, or signs of dehydration), or impossible to assess (eg, ear examination or abdominal examination), explaining why in-person assessments outperformed virtual ones in our study. Similarly, children and their families have specific information and emotional needs that are perhaps not addressed via virtual encounters. For example, caregivers may not have the reassurance they need from an encounter in which there was no physical examination and seek a second opinion in person in the ED.^31,32,33,34,35,36^ Literature has shown that physical examination is a common parental expectation and can reduce anxiety,^33^ potentially explaining our findings.

Identifying and understanding the needs that children and families experience during sick visits and establishing whether they can be met using virtual care modalities and technology will be important next steps in developing policies and infrastructure to support virtual care in children and establishing the scope of encounters for which virtual care is effective in children. Conversely, given current primary care shortages in Ontario (reflected in this cohort), it will be important to balance the relatively low clinical risk of increased ED use against the fact that virtual modalities may increase access to primary care practitioners, and that many patients and practitioners value this modality,^8,9,10,11,12,13,14^ when developing virtual care policies for children.

There were no differences in subsequent ED visits for children aged less than 3 months seen virtually or in person in our study. This could be because of the increased risk of serious illness in this age group. For example, clinical practice guideline recommendations favor emergent blood and urine testing for febrile infants,^37,38^ and these children would have been referred to the ED regardless of visit modality. Similarly, neonates with jaundice and feeding issues often require bilirubin testing, which can be difficult to obtain promptly in primary care settings.^39,40^ Separately, it may be that primary care practitioners are less comfortable relying on their clinical skills and physical examination in this age group due to a lack of exposure in training and in practice. This may decrease the advantage of in-person assessments relative to virtual ones, and lead to similar rates of subsequent ED use in this younger age group. Overall, primary care visits remain an important triaging tool for acutely ill young infants but visit modality may be less relevant in this age group.

### Limitations

This study has limitations. First, when defining the cohort, we chose to select 1 random sick episode per child, to avoid the overrepresentation in our sample of children with multiple sick episodes over the study period. Children with increased health care use may be different from those with lower utilization (eg, sicker, more medical anxiety) and this may have impacted our results. Second, when establishing exposure status, when children had multiple sick visits within an illness episode, we assigned an in-person exposure if any visit was in person. Multiple visits within an illness episode may represent illness progression and therefore assigning these as in person could underestimate our effect size estimates. Third, there may be unmeasured confounding related to patient (eg, SARS-CoV-2 anxiety) or practitioner (eg, SARS-CoV-2 anxiety, personal protective equipment availability, changes in billing infrastructure) preference for and use of virtual or in-person care over time. Children seen virtually were more likely to have a UPC, which provides the advantage of care continuity and preventative health and reduces the risk for disease progression or subsequent admissions. This would have biased our findings toward the null hypothesis. Conversely, families with access to a UPC may be different than families without (eg, racial, financial, health literacy, access to transportation, communication, and work or family responsibility differences),^41^ thus other unmeasured confounding factors may be at play. Fourth, our definitions for UPC were based on a 4-year look-back period. Since visits to primary care decreased during the pandemic,^42^ it is possible that children with UPCs may have been wrongly assigned to the no UPC group. This, too, would have underestimated the size of our estimates. Due to administrative family physician enrollment delays, 70% of infants aged less than 3 months in our cohort were not assigned a UPC at the time of their sick visit. This number may be an overestimation, which may have affected our models for this age group. Lastly, we were unable to capture sick visits not billed through the Ontario Health Insurance Plan (eg, visits conducted at community health centers or nurse-led clinics). Thus, our findings may not apply to these clinical contexts, commonly found in remote settings.

## Conclusions

In this population-based cohort study of sick visits to primary care practitioners, we found that children were more likely to visit the ED after a virtual visit than an in-person visit, but generally these visits were for low-acuity problems. While virtual care may sufficiently and safely triage visits that are unlikely to need admission or result in death, health system administrators and clinicians should consider the unique needs of children when developing policies and infrastructure around virtual care and when determining the modality of clinical encounters.

## Notes

Supplement 1. **eTable 1.** Data sources and variables used in the study **eTable 2.** List of outpatient sick child visit codes, well-child and immunizations primary care visit codes from the Corporate Provider Database (CPDB), and the mental health and addictions-related outpatient services from the Ontario Health Insurance Plan (OHIP) **eFigure.** Visual representation of the study timelines for data collection in relation to sick child visits to primary care providers in Ontario between September 2020 and March 2024 for (A) children with a single outpatient sick child visit during the study period, and (B) children with multiple sick child visits during the study period **eTable 3.** List of complex chronic condition diagnostic codes based on the International Classification of Disease – 10th edition – Canadian version (ICD-10-CA) **eTable 4.** Adjusted risk ratios and 95% confidence intervals for any emergency department visit by children following a visit to a primary care provider for an outpatient sick episode in Ontario, Canada, between September 1, 2020 and March 31, 2024, limiting to children with only one outpatient visit in their sick child episode **eTable 5.** Adjusted risk ratios and 95% confidence intervals for any emergency department visit by children following a visit to a primary care provider for an outpatient sick episode in Ontario, Canada, between September 1, 2020 and March 31, 2024, defining visit modality based on first outpatient visit only

Supplement 2.
Data Sharing Statement
