# Supplementary material for: Virtual Care Provision and Emergency Department Use Among Children and Youth
Source: JAMA Netw Open. 2025 Dec 18;8(12):e2550532. doi: 10.1001/jamanetworkopen.2025.50532 (PMC12715653; doi:10.1001/jamanetworkopen.2025.50532)
Supplement: Supplement 1. — eTable 1. Data sources and variables used in the study eTable 2. List of outpatient sick child visit codes, well-child and immunizations primary care visit codes from the Corporate Provider Database (CPDB), and the mental health and addictions-related outpatient services from the Ontario Health Insurance Plan (OHIP) eFigure. Visual representation of the study timelines for data collection in relation to sick child visits to primary care providers in Ontario between September 2020 and March 2024 for (A) children with a single outpatient sick child visit during the study period, and (B) children with multiple sick child visits during the study period eTable 3. List of complex chronic condition diagnostic codes based on the International Classification of Disease – 10th edition – Canadian version (ICD-10-CA) eTable 4. Adjusted risk ratios and 95% confidence intervals for any emergency department visit by children following a visit to a primary care provider for an outpatient sick episode in Ontario, Canada, between September 1, 2020 and March 31, 2024, limiting to children with only one outpatient visit in their sick child episode eTable 5. Adjusted risk ratios and 95% confidence intervals for any emergency department visit by children following a visit to a primary care provider for an outpatient sick episode in Ontario, Canada, between September 1, 2020 and March 31, 2024, defining visit modality based on first outpatient visit only [file jamanetwopen-e2550532-s001.pdf]

Supplemental Online Content

Freire G, Cohen E, Stukel TA, et al. Virtual care provision and emergency department use among Ontario children and youth. *JAMA Netw Open.* 2025;8(12):e2550532. doi:10.1001/jamanetworkopen.2025.50532

eTable 1. Data sources and variables used in the study
eTable 2. List of outpatient sick child visit codes, well-child and immunizations primary care visit codes from the Corporate Provider Database (CPDB), and the mental health and addictions-related outpatient services from the Ontario Health Insurance Plan (OHIP)
eFigure. Visual representation of the study timelines for data collection in relation to sick child visits to primary care providers in Ontario between September 2020 and March 2024 for (A) children with a single outpatient sick child visit during the study period, and (B) children with multiple sick child visits during the study period
eTable 3. List of complex chronic condition diagnostic codes based on the International Classification of Disease – 10th edition – Canadian version (ICD-10-CA)
eTable 4. Adjusted risk ratios and 95% confidence intervals for any emergency department visit by children following a visit to a primary care provider for an outpatient sick episode in Ontario, Canada, between September 1, 2020 and March 31, 2024, limiting to children with only one outpatient visit in their sick child episode
eTable 5. Adjusted risk ratios and 95% confidence intervals for any emergency department visit by children following a visit to a primary care provider for an outpatient sick episode in Ontario, Canada, between September 1, 2020 and March 31, 2024, defining visit modality based on first outpatient visit only

This supplemental material has been provided by the authors to give readers additional information about their work.

© 2025 Freire G et al. *JAMA Network Open*.

eTable 1. Data sources and variables used in the study

Data Source	Variables
Ontario Health Insurance Plan (OHIP)	Billing codes to identify sick visits, mental health visits, well-child visits (see eTable 2)
National Ambulatory Care Reporting System (NACRS)	ED visits, CTAS score, ED discharge diagnosis, date of ED visit
Canadian Institute for Health Information's Discharge Abstract Database (CIHI-DAD)	Hospital admission, chronic complex conditions
Corporate Provider Database (CPDB)	New, occasional, and usual provider of care
Client Agency Program Enrolment (CAPE)	Provider enrollment model
Registered Persons Database (RPDB)	Age, sex
Ontario Marginalization Index (ON-Marg)	Material resources quintile
Canadian Census 2016	Rurality
Postal Code Conversion File (PCCF)	Rurality

Abbreviations: CAPE, Client Agency Program Enrolment; CIHI, Canadian Institute for Health Information's; CPDB, Corporate Provider Database; CTAS, Canadian Triage and Acuity Scale; DAD, Discharge Abstract Database; ED, emergency department; NACRS, National Ambulatory Care Reporting System; OHIP, Ontario Health Insurance Plan; ON-Marg, Ontario Marginalization Index; PCCF, Postal Code Conversion File; RPDB, Registered Persons Database

© 2025 Freire G et al. *JAMA Network Open*.

eTable 2. List of outpatient sick child visit codes, well-child and immunizations primary care visit codes from the Corporate Provider Database (CPDB), and the mental health and addictions-related outpatient services from the Ontario Health Insurance Plan (OHIP)

Description	Diagnostic or Fee Codes
**Outpatient Sick Child Visits**	
In-Person	A001, A003, A004, A005, A006, A007, A008, A900, A901, A902 A905, A911, A912-, A261, A262, A263, A264, A265, A260, A662, A661
Virtual	B099, B100, B200, B103, B203, B209, K080, K081, K082, K083 or any location code = P
**Well Child and Immunization Primary Care Visits**	
Annual health exam-child after 2nd birthday	K017
Annual health exam-child-after 2nd birthday paediatric	K267
Annual health exam-paediatrics-adolescent-office	K269
18 month well baby check – family physician or generalist	A002
18 month well baby check – pediatrician	A268
Any diagnostic code with 916 or 917 (well baby care)	Any of above fee codes with diagnostic code 916 or 917
D./T. proc.-injections-intradermal/muscular etc. ea. add	G372
D./T. proc.-inj. intradermal/musc. basic fee (shick test)	G373
D&T immunization-with visit, each inject.	G538
D&T immunization-sole reason, first injection	G539
Influenza agent + visit	G590
Influenza agent sole reason	G591
DTaPIPV-Diphtheria, Tetanus, acellular Pertussis, Inactivated Polio Virus paediatric	G840
DTaPIPVHib-Diphtheria, Tetanus, acellular Pertussis, Inactivated Polio Virus, Haemophilus influenza type b paediatric	G841
MenCC-Meningococcal C Conjugate	G844
MMR - Measles, Mumps, Rubella	G845
Pneu – Pneumococcal Conjugate	G846
Var-Varicella	G848
**Mental Health Related Outpatient Primary Care Visits**	
	291-292, 295-299, 300-304, 306-307, 309, 311, 313-315, 897-902, 904-906, 909

© 2025 Freire G et al. *JAMA Network Open*.

eFigure. Visual representation of the study timelines for data collection in relation to sick child visits to primary care providers in Ontario between September 2020 and March 2024 for (A) children with a single outpatient sick child visit during the study period, and (B) children with multiple sick child visits during the study period

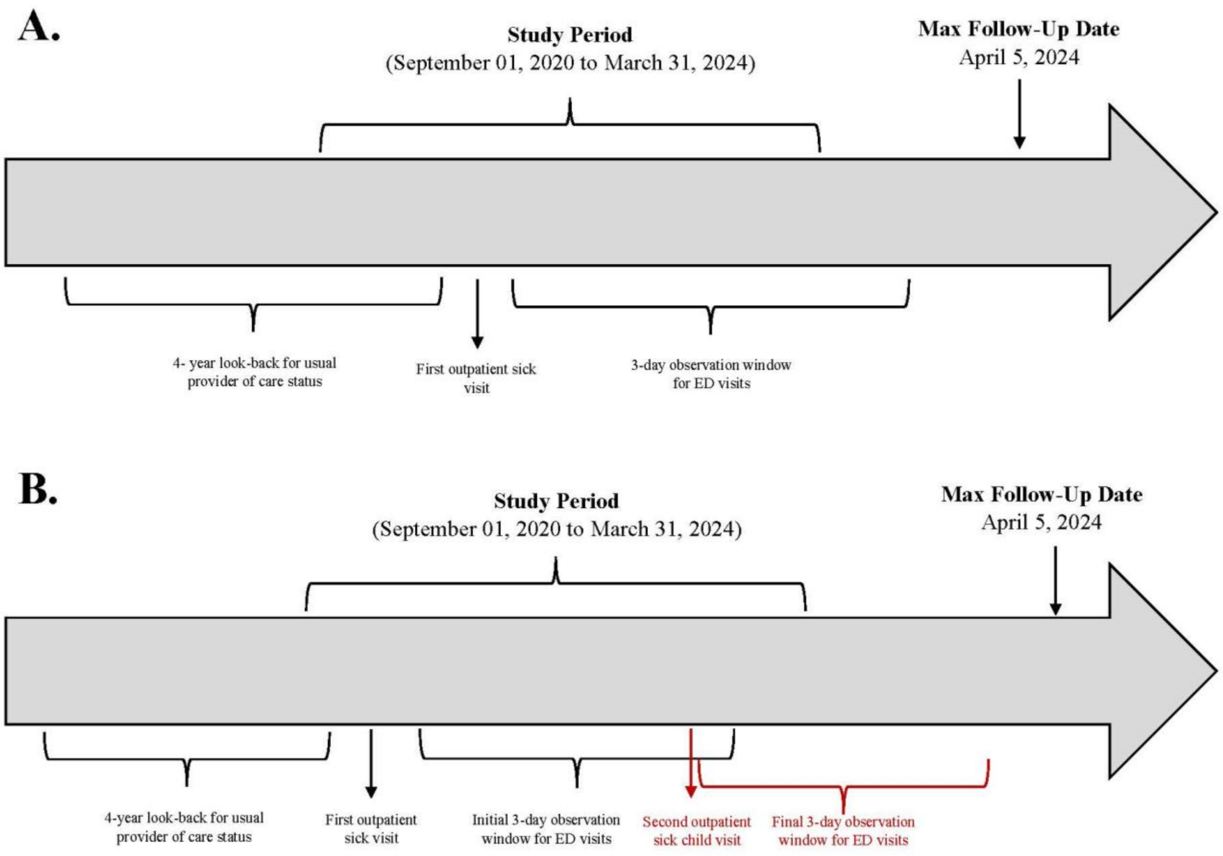

© 2025 Freire G et al. *JAMA Network Open*.

eTable 3. List of complex chronic condition diagnostic codes based on the International Classification of Disease – 10th edition – Canadian version (ICD-10-CA)

Categories	Subcategories	ICD-10-CA
**Neurologic and Neuromuscular**	Brain and spinal cord malformations	Q00-Q07, G90.1
	Mental retardation	F71-F73
	CNS degeneration and diseases	E75.0, E75.1, E75.2, E75.4, F84.2, G11.1–G11.4, G11.8, G11.9, G12.0–G12.2, G12.8, G12.9, G31.00, G31.02, G31.8, G32.8, G93.8, G93.9, G94, G91.1, G31.9, G25.3, G95.1, G95.8, G90.9, Q85.1
	Infantile cerebral palsy	G80
	Epilepsy	G40.1, G40.2, G40.3, G40.4, G40.81, G40.91
	Other disorders of CNS	G37.1, G37.2, G37.8, G81.99, G82.5, G83.5, G83.9, G93.1, G93.5, R40.20
	Occlusion of cerebral arteries	I63.3, I63.5
	Muscular dystrophies and myopathies	G71, G72
	Movement diseases	G10, G20, G21.0, G21.1, G21.8, G23.0-G23.2, G23.8, G24.0, G24.8, G25.3-G25.5, G25.8, G25.9, G80.3
	Devices (Mechanical complication of nervous system or cerebrospinal fluid drainage device)	T85.0, T85.1, T85.7, Z98.2, Z45.8
**Cardiovascular**	Heart and great vessel malformations	Q20, Q21.2-Q24, Q25.1-Q25.9, Q26, Q28.2, Q28.3, Q28.9
	Endocardium diseases	I34.0, I34.8, I36.0, I36.8, I37.0, I37.8
	Cardiomyopathies	I42, I43, I51.5
	Conduction disorder	I44, I45, I47, I48, I49.0
	Dysrhythmias	I49.1-I49.5, I49.8, I49.9, R00.1
	Other	I27.0, I27.1, I27.2, I27.8, I27.9, I50.9, I51.7, I42.8, I63.1, I63.2, Z95.1
	Devices (Mechanical complication of cardiac device implant and graft)	T82.0-T82.1, T82.2, T82.5,T82.6, T82.7, Z95.0, Z95.2, Z95.3, Z95.8, Z45.00, Z45.01, Z45.08, Z95.9
	Transplantation	T86.200, T86.201, Z94.1

© 2025 Freire G et al. *JAMA Network Open*.

Categories	Subcategories	ICD-10-CA
**Respiratory**	Respiratory malformations	Q30-Q34, P280
	Chronic respiratory diseases	G47.31, I26.9, J84.1, J96.0, J96.1, Z90.2
	Cystic fibrosis	E84
	Other	N/A
	Devices (Tracheostomy complications)	J95.00–J95.03, J95.08, Z43.0, Z93.0, Z99.0, T85.6, Z99.1
	Transplantation	T86.800, T86.801, Z94.2
**Renal and Urologic**	Congenital anomalies	Q60-Q64
	Chronic renal failure	N18
	Other	Z90.5, Z90.6
	Chronic bladder diseases	G83.4, N31.2, N31.9
	Devices (Cystostomy or urinostomy)	T85.7, Z93.5, Z93.6, Z91.1, Z99.2, Z43.5, Z43.6, Z46.6
	Transplantation	T86.100, T86.101, Z94.0
**Gastrointestinal**	Congenital anomalies	Q39.0-Q39.4, Q41-Q45
	Chronic liver disease and cirrhosis	K73, K74, K75.4, K76.0-K76.3, K76.5, K76.8
	Inflammatory bowel diseases	K50, K51
	Other	I82.0, K55.1, K56.2, K59.3, Z98.0, Z90.3, Z90.4
	Devices (Gastrostomy, enterostomy, fitting and adjustment of intestinal appliance and device)	K91.62, K91.61, Z93.1–Z93.4,Z43.1–Z43.4, Z46.8
	Transplantation	T86.400, T86.401, T86.880, T86.881, Z94.4, Z94.81, Z94.82
**Hematologic or immunologic**	Hereditary anemias	D55-D58
	Aplastic anemias	D60, D61, D71
	Hereditary immunodeficiency	D80-D89, D72.0, M30.3, M35.9
	Coagulation/hemorrhagic	D66, D68.2, D69.30, D69.4
	Leukopenia	D70.0
	Hemophagocytic Syndromes	D76.1-D76.3
	Sarcoidosis	D86.9
	Acquired immunodeficiency	B24
	Polyarteritis nodosa and related conditions	M30.0, M31.0, M31.1, M31.30, M31.4, M31.6
	Diffuse diseases of connective tissue	M32.9, M33.9, M34.0, M34.1, M34.9
	Other	N/A
	Transplantation	N/A

© 2025 Freire G et al. *JAMA Network Open*.

Categories	Subcategories	ICD-10-CA
**Metabolic**	Amino acid metabolism	E70.0, E70.2, E70.3, E70.8, E71.0–E71.3, E72.0–E72.4, E72.8, E72.9
	Carbohydrate metabolism	E74
	Lipid metabolism	E75, E77.0, E77.1, E78, E88.1, E88.8
	Storage disorder	E76.0-E76.3, E85
	Other metabolic disorders	E79.1, E79.8, E80.4–E80.7, E83.0, E83.1, E83.3, E83.4, D84.1, E88, H49.8
	Endocrine disorders	E00.9, E22.2, E23.0, E23.2, E23.3, E23.7, E24.0, E24.2, E24.3, E24.8, E24.9, E25.0, E25.8, E25.9, E26.8
	Devices (Endocrine implants)	Z96.4
**Other Congenital or Genetic Defect**	Chromosomal anomalies	Q90.9, Q91.3, Q91.4, Q91.7, Q92.8, Q93, Q95.0, Q96.9, Q97, Q98, Q99.8, Q99.9
	Bone and join anomalies	E34.3, M41.0, M41.2, M41.39, M41.8, M41.9, M43.3, M96.5, Q72.2, Q75.0, Q75.2, Q75.9, Q76.0–Q76.2, Q76.4–Q76.7, Q77, Q78.0–Q78.4
	Diaphragm and abdominal wall	K44.9, Q79.0-Q79.5, Q79.9
	Other congenital anomalies	Q81, Q87.1-Q87.3, Q87.40, Q87.8, Q89.7, Q89.9, Q99.2
**Neoplasma**	Neoplasms	C00–C97, D01–D09, D36.7, D10–D36, D37–D48, Q85.0
	Transplantation	T86.000, T86.001, Z94.80, Z94.83
**Miscellaneous, Not Elsewhere Classified**	Devices (Mechanical complication of internal orthopedic device implant and graft)	T84.09, T84.8, T84.19, T84.4, T84.59, T84.69, T84.7, T87.09–, T87.19, T87.2, Y83.1, Y83.3, Z99.8
	Transplantation	T86.88–T86.9

Abbreviations: CNS, Central Nervous System; ICD-10-CA, International Statistical Classification of Diseases and Related Health Problems 10th Revision

© 2025 Freire G et al. *JAMA Network Open*.

eTable 4. Adjusted risk ratios and 95% confidence intervals for any emergency department visit by children following a visit to a primary care provider for an outpatient sick episode in Ontario, Canada, between September 1, 2020 and March 31, 2024, limiting to children with only one outpatient visit in their sick child episode.

Age category		< 3 months N=115,858			3 months to < 2 years N=269,553			2 to 17 years N=2,117,855		
		n (%)	Adjusted RR	95% CI	n (%)	Adjusted RR	95% CI	n (%)	Adjusted RR	95% CI
Any ED visit	In-person	2,854 (2.7)	1.00	NA	6,971 (3.3)	1.00	N/A	34,789 (2.3)	1.00	NA
	Virtual	305 (2.8)	1.16	1.02-1.32	2,630 (4.3)	1.49	1.41-1.57	13,079 (2.1)	1.20	1.15-1.24

Abbreviations: CI, Confidence Interval; ED, emergency department; NA, Not Applicable; RR, risk ratio

All models adjusted for sex, presence of a complex chronic condition, material resource quintile, rurality, whether the index provider saw the child in the previous 2 years, type of usual provider of care, and age at the index visit (for the 2-17 years group only).

© 2025 Freire G et al. *JAMA Network Open*.

eTable 5. Adjusted risk ratios and 95% confidence intervals for any emergency department visit by children following a visit to a primary care provider for an outpatient sick episode in Ontario, Canada, between September 1, 2020 and March 31, 2024, defining visit modality based on first outpatient visit only

Age category		< 3 months N=132,352			3 months to < 2 years N=282,720			2 to 17 years N=2,193,431		
		n (%)	Adjusted RR	95% CI	n (%)	Adjusted RR	95% CI	n (%)	Adjusted RR	95% CI
Any ED visit	In-person	3,283 (2.7)	1.00	NA	7,491 (3.5)	1.00	NA	37,041 (2.4)	1.00	NA
	Virtual	384 (3.2)	1.24	1.10-1.39	2,996 (4.5)	1.50	1.42-1.58	14,853 (2.2)	1.22	1.18-1.26

Abbreviations: CI, Confidence Interval; ED, emergency department; NA, Not Applicable; RR, risk ratio

All models adjusted for sex, presence of a complex chronic condition, material resource quintile, rurality, whether the index provider saw the child in the previous 2 years, type of usual provider of care, number of outpatient visits in the index sick episode, and age at the index visit (for the 2-17 years group only).

© 2025 Freire G et al. *JAMA Network Open*.
